# Comparative Study of Human and Murine Aortic Biomechanics and Hemodynamics in Vascular Aging

**DOI:** 10.3389/fphys.2021.746796

**Published:** 2021-10-25

**Authors:** Sara E. Hopper, Federica Cuomo, Jacopo Ferruzzi, Nicholas S. Burris, Sara Roccabianca, Jay D. Humphrey, C. Alberto Figueroa

**Affiliations:** ^1^Department of Biomedical Engineering, University of Michigan, Ann Arbor, MI, United States; ^2^Department of Bioengineering, The University of Texas at Dallas, Richardson, TX, United States; ^3^Department of Radiology, University of Michigan, Ann Arbor, MI, United States; ^4^Department of Mechanical Engineering, Michigan State University, East Lansing, MI, United States; ^5^Department of Biomedical Engineering, Yale University, New Haven, CT, United States; ^6^Vascular Biology and Therapeutics Program, Yale University, New Haven, CT, United States; ^7^Department of Surgery, University of Michigan, Ann Arbor, MI, United States

**Keywords:** arterial stiffness, pulse wave velocity (PWV), fluid-solid-interaction, hypertension, aortic morphology

## Abstract

**Introduction:** Aging has many effects on the cardiovascular system, including changes in structure (aortic composition, and thus stiffening) and function (increased proximal blood pressure, and thus cardiac afterload). Mouse models are often used to gain insight into vascular aging and mechanisms of disease as they allow invasive assessments that are impractical in humans. Translation of results from murine models to humans can be limited, however, due to species-specific anatomical, biomechanical, and hemodynamic differences. In this study, we built fluid-solid-interaction (FSI) models of the aorta, informed by biomechanical and imaging data, to compare wall mechanics and hemodynamics in humans and mice at two equivalent ages: young and older adults.

**Methods:** For the humans, 3-D computational models were created using wall property data from the literature as well as patient-specific magnetic resonance imaging (MRI) and non-invasive hemodynamic data; for the mice, comparable models were created using population-based properties and hemodynamics as well as subject-specific anatomies. Global aortic hemodynamics and wall stiffness were compared between humans and mice across age groups.

**Results:** For young adult subjects, we found differences between species in pulse pressure amplification, compliance and resistance distribution, and aortic stiffness gradient. We also found differences in response to aging between species. Generally, the human spatial gradients of stiffness and pulse pressure across the aorta diminished with age, while they increased for the mice.

**Conclusion:** These results highlight key differences in vascular aging between human and mice, and it is important to acknowledge these when using mouse models for cardiovascular research.

## Introduction

Aging is a primary risk factor for numerous cardiovascular diseases (Lakatta et al., 2009; Safar, 2010). Even in the absence of co-morbidities that often arise in aging, natural aging-induced changes in structure and function of the vascular system include pronounced central artery stiffening and associated changes in the peripheral vasculature (Mitchell, 2009; Humphrey et al., 2016). Such structural stiffening results in large part from changes in the composition and thickness of the arterial wall: elastin fibers undergo fatigue-related damage and can begin to degrade naturally despite their long half-life, while proteoglycans and collagen fibers can both remodel and accumulate, also influenced by increased cross-linking (Greenwald, 2007). Whereas loss of elastic fiber integrity can reduce central artery resilience, and thus overall biomechanical functionality, the overall increase in structural stiffness due to accumulating extracellular matrix can compromise hemodynamics, and thus vascular physiology, via increases in the speed at which the pulse pressure travels along the central vessels (Mitchell, 2009; Safar et al., 2020). In particular, this structural stiffening (or loss of compliance) compromises the dampening function of the central arteries, resulting in both augmented central pulse pressures that adversely affect the heart and a deeper penetration of pulsatility into the peripheral vessels that affects end-organ function (Mitchell, 2008, 2009; Boutouyrie et al., 2021).

Direct comprehensive measurement of aortic stiffness, both material and structural, is not possible *in vivo*. Rather, the gold standard method to estimate the structural stiffness of the aorta clinically is to measure the pulse wave velocity (PWV). PWV increases with natural aging as well as with many related co-morbidities and has been linked clinically to hypertension and other cardiovascular diseases (Laurent et al., 2006; Laurent and Boutouyrie, 2015; Townsend et al., 2015). Connecting stiffening to underlying histo-mechanical changes in the vessel wall is difficult in a clinical setting, hence many have turned to mouse models to obtain more information about mechanisms of arterial stiffening and its relation to other conditions, including hypertension (Fleenor et al., 2010; Rammos et al., 2014; Lerman et al., 2019). Benefits of mouse models include rapid maturation and aging, ability of genetic manipulation, low cost, and enhanced experimental control, particularly in biomechanical measurement and computation (Ferruzzi et al., 2013; Cuomo et al., 2015, 2019).

No mouse model phenocopies the human cardiovascular system exactly, hence results should be synthesized across multiple studies of different mouse models and interpreted carefully (Ferruzzi et al., 2016). Obvious differences between humans and mice include their very different lifespans [70+ years versus 2+ years (Hamlin and Altschuld, 2011)] and body size, with adult mice weighing approximately 0.05% that of the adult human (Doevendans et al., 1998). Cardiovascular differences include cardiac output [5 L/min for humans (Brandfonbrener et al., 1955), 15 mL/min for mice (Doevendans et al., 1998)], which reflects the size difference, but also heart rate [60 bpm for humans, 600 bpm for mice (Papadimitriou et al., 2008)]. Blood pressure is yet similar between the species. Notwithstanding the order of magnitude higher heart rate in mice, the expected number of cardiac cycles over a lifetime is still higher in humans (2.2 × 10^9^) than in mice (6.3 × 10^8^), which is expected to impact mechanical fatigue-induced loss of elastic fiber integrity (Greenwald, 2007). Indeed, given that the normal half-life of vascular elastin is many decades (Davis, 1993), it appears that arterial aging in mice can be attributed more to the accumulation of proteoglycans and remodeled collagen (Fleenor et al., 2010; Ferruzzi et al., 2018) than to the loss of elastic fiber integrity that occurs in humans alongside matrix remodeling. It is for this reason that different mouse models of compromised elastic fiber integrity can be useful in arterial aging research (Ferruzzi et al., 2016). Further differences between human and murine vascular aging remain to be established.

Computational simulations, particularly those based on fluid-solid-interactions (FSI), can be used to quantify and compare hemodynamic quantities that are difficult to infer experimentally in humans and mice. In this study, FSI models were built for young and old female adult humans and mice to compare multiple hemodynamic and biomechanical effects of aging on the two species. For the humans, representative computational models for young and old adult subjects were defined using imaging data, non-invasive pressure measurements, and population-specific arterial wall properties. For the mice, representative population-based computational models for young and old adults were obtained using *in vivo* and *in vitro* data on vascular anatomy, hemodynamics, and wall mechanics. Both human and mouse subjects were considered healthy, with no comorbidities, with the ages between species generally equivalent. Structural and hemodynamic results were compared to determine the species- and age-related differences between subjects.

## Materials and Methods

In setting an age correspondence between mice and humans, the Jax Mice website^1^ suggests that a 13–27 weeks old mouse is similar to a 20–30 years old human, and an 81–108 weeks old mouse is similar to a 56–69 years old human. Imaging and brachial cuff pressure data were obtained for young adult (31 years old) and older adult (81 years old) female human subjects. This study was approved by the University of Michigan Board of Review (HUM00041514). *In vivo* imaging and *ex vivo* biomechanical testing data were obtained for young adult (20 weeks old) and older adult (100 weeks old) female mice. All animal procedures were approved by the Institutional Animal Care and Use Committee (IACUC) of Yale University. The mice had a mixed C57BL/6 × 129/SvEv background, generated as *Fbln5^+/+^* by breeding *Fbln5^+/–^* heterozygous pairs (Yanagisawa et al., 2002). For the young adult mice, data were collected for three cohorts at 20 weeks of age: one for anatomy, one for hemodynamics, and one for wall mechanics. For the old adult mice, data were collected from two cohorts that aged naturally to 100 weeks: one for anatomy and one for both hemodynamics and wall mechanics.

### Experimental Methods

#### Vascular Anatomy

Magnetic resonance angiography (MRA) was performed on human subjects at the University of Michigan Medical Center. These exams were performed on 3T MRI scanners (Ingenia, Philips, Best, Netherlands) using a 32-channel torso coil. A non-contrast MRA was performed spanning the thoraco-abdominal aorta using a 3D balanced turbo field echo sequence with navigator-based respiratory compensation (TE: 1.3 ms, TR: 4.3 ms, resolution: 0.7 × 0.7 × 1.5 mm).

As described previously (Cuomo et al., 2019), mice were anesthetized with 1–2% isoflurane and given a bolus intravenous (jugular vein) injection of nanoemulsion formulation (Fenestra VC, MediLumine Inc., Montreal, QC, Canada), at a dose of 0.2 ml/20 g, as a blood-pool contrast agent for prolonged vascular imaging. The animal was immediately placed prone in a micro-CT scanner (eXplore CT120, GE healthcare) for non-gated whole-body scanning. Images were reconstructed as isotropic 49 μm × 49 μm × 49 μm voxels. A relatively constant heart rate (±10%) was achieved by careful maintenance of isoflurane anesthesia and body temperature.

#### Hemodynamics

40-phase, 2D phase-contrast MRI (PC-MRI) images were acquired in planes orthogonal to the human aorta at the levels of the mid-ascending aorta and distal descending aorta at the diaphragm (TE: 3.0 ms, TR: 5.0 ms, slice thickness: 8 mm, temporal resolution: 19 ms, velocity encoding value: 150 cm/s). Brachial artery blood pressure was measured with a standard non-invasive cuff method, averaged from patient data dating back 2 years (averaged values of 111/66 mmHg for young adult and 111/55 mmHg for old adult). It is important to note that the older human was considered healthy and free of cardiovascular diseases and medication, except for long-term, low-dose diuretic (hydrochlorothiazide) to control blood pressure which resulted in a reduction of blood pressure from 140/85 mmHg prior to medication to the 111/55 mmHg used in this study.

As described previously (Ferruzzi et al., 2018; Cuomo et al., 2019), mice were laid supine after isoflurane inhalation anesthesia (2–3% for induction, 1.5% for maintenance) and secured on a surgical platform with a recirculating heating pad (TP-500 Heat Therapy Pump, Gaymar Industries Inc., Orchard Park, NY, United States) to maintain body temperature at 37°C. Mean blood velocity and luminal diameters were then acquired via ultrasound (Vevo 2100 system, FUJIFILM VisualSonics) in the ascending thoracic aorta (ATA), infrarenal abdominal aorta (IAA), and a common carotid artery (CCA). Cardiac output (CO) was measured with standard B-mode transthoracic echocardiography and central aortic pressure was measured using a SPR-1000 Millar pressure catheter with a diameter of 1F. Hemodynamic measurements were performed on *n* = 10 young adult and *n* = 10 old adult mice.

#### Aortic Biomechanics

It is the *structural* stiffness, the product of the intrinsic *material* properties and wall thickness, which dictates the hemodynamic response to an artery under pulsatile flow. A four-fiber family constitutive model was used to quantify the biaxial *material* stiffness of the arterial wall, both human and murine. This model contains 8 parameters to capture the macroscopic behavior resulting from the contributions of elastic fibers, collagen fibers, and additional matrix (Ferruzzi et al., 2011, 2013). The strain energy density equation for the four-fiber family model can be seen in Eq. 1.


(1) $$W\,\left(C,M^{i}\right)=\frac{c}{2}\,\left(I_{c}-3\right)+\sum_{i=1}^{4}\frac{c_{1}^{i}}{4\,c_{2}^{i}}\,\left\{\exp\left[c_{2}^{i}\,{\left(I\,V_{c}^{i}-1\right)}^{2}\right]-1\right\}$$

Where ***C*** is the right Cauchy-Green tensor, *F* is the deformation tensor [*F* = *diag*(λ_*r*_,λ_ϑ_,λ_*z*_)],*M*^*i*^ represents the orientation of the fiber families [$M^{i}=\left(0,\sin\alpha_{0}^{i},\cos\alpha_{0}^{i}\right)$], and $I\,V_{C}^{i}=\lambda_{\vartheta }^{2}\,\sin^{2}\alpha_{0}^{i}+\lambda_{z}^{2}\,\cos^{2}\alpha_{0}^{i}$. The 8-material parameters fit for each aortic region are *c, $c_{1}^{1}$ $c_{2}^{1}$, $c_{1}^{2}$*, *$c_{2}^{2}$*, *$c_{1}^{3,4}$*, *$c_{2}^{3,4}$*, and *α*_0_, where *c* refers to the material parameter for the elastin mass fraction and *$c_{1}^{1}$ $c_{2}^{1}$* and *$c_{1}^{2}$*, *$c_{2}^{2}$* refer to the collagen coefficients for the axial and circumferential fiber families, respectively. *$c_{1}^{3,4}$*, *$c_{2}^{3,4}$*, and *α*_0_ refer to the material parameters and angle for symmetric diagonal collagen fiber families.

**FIGURE 1:**
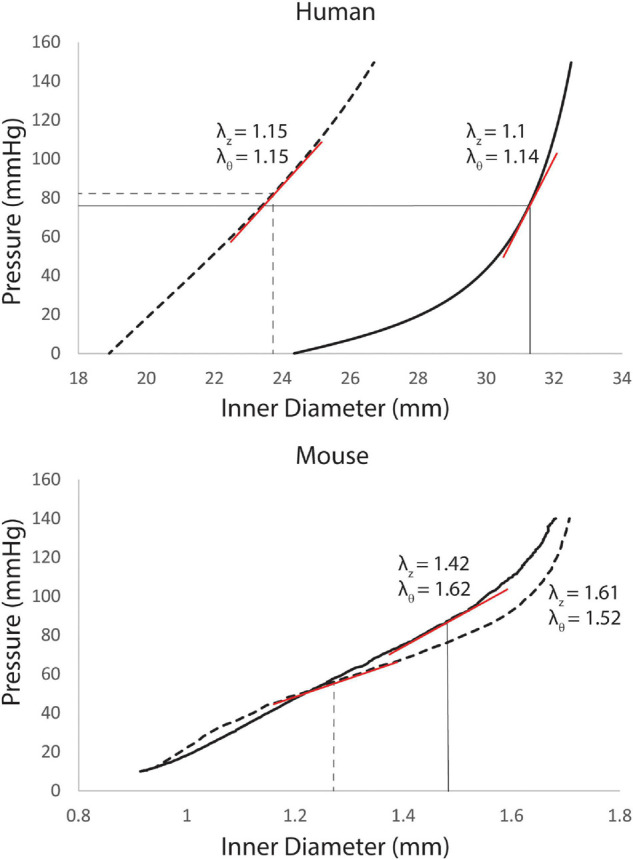
Pressure-diameter curves for the ascending thoracic aorta (ATA) of young (dashed line) and old (solid lines) adult subjects with corresponding values of *in vivo* axial stretch (λ_*z*_, from literature data) and circumferential stretch (λ_θ_, iteratively calibrated). Linearized stiffness (red lines) were used in our FSI simulations. For the humans (top), curves are recreated from best-fit values of the 8 material properties from Roccabianca et al. (2014) with *in vivo* circumferential stretches adjusted until *in vivo* mean pressures corresponded with *in vivo* mean diameters. For the mice (bottom), the pressure-diameter curves were measured directly *in vitro*, with best-fit values of the material parameters determined via non-linear regression of the biaxial data.

For the humans, the 8 model parameters and *in vivo* axial stretch (λ_*z*_) were specified from literature data for three segments of the aorta: ATA, descending thoracic aorta (DTA), and IAA for different age groups (Roccabianca et al., 2014). Material parameters for the young adult were based on data for 31–60 years old subjects, while those for the old adult were based on data for the 61+ years old age group. An iterative approach was then used to adjust the resulting pressure-diameter curves to match patient data on *in vivo* pressure and diameter. The unloaded diameter [and therefore the *in vivo* circumferential stretch (λ_θ_)] was iteratively adjusted until the mean pressure [estimated as *P*_*mean*_ = (2*P*_*dias*_ + *P*_*sys*_)/3 from cuff measurements] corresponded with the *in vivo* mean diameter [estimated as *D*_*mean*_ = (2*D*_*dias*_ + *D*_*sys*_)/3 from PC-MRI images] on the pressure diameter curve (Roccabianca et al., 2014). Examples of adjusted pressure-diameter curves can be seen in Figure 1.

For the mice, *in vitro* mechanical testing was performed on four excised segments of the murine aorta [ATA, DTA, suprarenal abdominal aorta (SAA), and IAA] and a CCA with a computer-controlled custom biaxial testing device (Gleason et al., 2004), as described previously (Cuomo et al., 2019). After standard preconditioning, seven cyclic pressure diameter tests were performed: cyclic pressure diameter tests from 10 to 140 mmHg at three different fixed values of axial stretch (λ_*z*_) (95, 100, and 105% of the *in vivo* value) and cyclic axial extension tests at four fixed values of transmural pressure (10, 60, 100, and 140 mmHg) (Ferruzzi et al., 2013). Best-fit values of the eight model parameters for the same four-fiber family constitutive model were determined for *n* = 5 young adult and *n* = 5 old adult mice (Ferruzzi et al., 2013).

### Computational Modeling

FSI models were built with the open source computational hemodynamics platform CRIMSON (Arthurs et al., 2021). Data from imaging, hemodynamics, and wall mechanics were combined to create FSI models comprised of 3D anatomical models having spatially varying anisotropic wall mechanical properties and external tissue support, with inlet flow waveforms and 3-element Windkessel models on each outflow branch.

#### Anatomical Models and Finite Element Meshes

The CRIMSON GUI was used to create 3D models of the aorta and main branches. Centerline paths were determined for each vessel of interest and circular contours were added perpendicular to the centerline with discrete spacing to represent the vessel lumen. 3D volumes resulted by lofting between contours and applying a union function to blend the aorta and main branching vessels. For the humans, the 3D representation was based on MRA images. For the mice, the 3D representation was based on μCTA images. Furthermore, for the mice, 9 sets of intercostal arteries were added based on the location of the ribs.

Field-driven mesh adaptation techniques were used to create finite-element meshes refined on regions of high velocity gradients. For the humans, meshes had 1.4 × 10^6^ tetrahedral elements and 2 × 10^5^ nodes for the young adult and 2.6 × 10^6^ tetrahedral elements and 5 × 10^5^ nodes for the old adult. For the mice, meshes had 1.7 × 10^6^ tetrahedral elements and 3 × 10^5^ nodes for the young adult and 2.9 × 10^6^ tetrahedral elements and 5 × 10^5^ nodes for the old adult.

#### Boundary Conditions

For the humans, available data on pressure and cardiac output for each subject were used to inform computational models. For the mice, allometric scaling (Cuomo et al., 2019) was used to incorporate data from hemodynamics and wall mechanics from multiple mouse cohorts in to the 3D FSI models. Briefly, allometric scaling took the form Υ = Υ_0_M^*b*^ with Υ being the quantity of interest (e.g., CO, resistance R_TOT_, and compliance C_TOT_), *Υ*_*0*_ the normalized quantity from experimental data, M the body mass, and *b* a scaling constant. Linear regression of log-log plots of the quantify of interest versus body mass was used to determine the coefficients (Cuomo et al., 2019).

##### Inflow boundary condition

**FIGURE 2:**
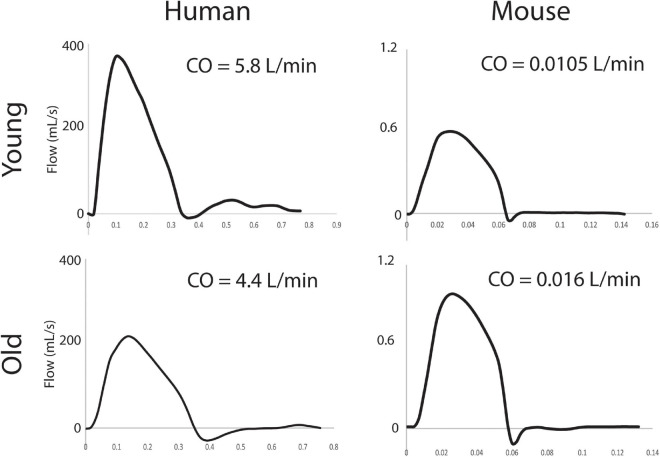
ATA inflow waveforms (mL/s) and associated values of CO (L/min).

For the humans, aortic inflow waveforms were generated from PC-MRI velocity and diameter data using the freely available Medviso software Segment, version 3.0 R8115 (Lee et al., 2008). A semi-automated technique was used to segment the ATA lumen over one cardiac cycle. For the mice, population-averaged flow waveforms from a hemodynamics cohort were used, as described previously (Cuomo et al., 2019). CO was allometrically scaled based on body mass for the old and young subjects, again as before (Cuomo et al., 2015, 2019). ATA flow waveforms and cardiac output can be seen in Figure 2 for all subjects.

##### Outflow boundary conditions

A three-element Windkessel model was applied to the outlet of all branches. First, total arterial resistance (R_TOT_) and total arterial compliance (C_TOT_) were estimated. R_TOT_ was estimated from P_*mean*/*CO*_ for both species. For the humans, C_TOT_ was estimated from (*Q_max_* − *Q_min_*/*P_systole_* − *P_diastole_*)^Δ*t*^ (Xiao et al., 2014). For the mice, C_TOT_ was estimated from the slope of the diastolic decay curve of the experimental ATA pressure waveforms [*P*_*diastole*_ (*t*) = *P*_0_ *exp* (−*t*/*R*_*TOT*_*C*_*TOT*_)] (Simon et al., 1979). Furthermore, in the mice, R_TOT_ and C_TOT_ were estimated using population data from the hemodynamics and wall mechanics cohort, then allometrically scaled as described previously.

R_TOT_ and C_TOT_ can be separated into 3D (central) and peripheral portions. The 3D portion is set by the anatomy and stiffness of the central vasculature; the peripheral portion can be estimated iteratively. Once R_TOT_ and the distal portion of C_TOT_ are obtained, they must be distributed among the outflow branches of each computational model to ultimately specify proximal resistance ($R_{p\,r\,o\,x}^{i}$), compliance (*C*^*i*^), and distal resistance ($R_{d\,i\,s\,t}^{i}$) for each outlet (*i*) as described previously (Cuomo et al., 2019).

#### Wall Mechanics

The FSI simulations were based on a coupled momentum formulation (Figueroa et al., 2006). The vessel walls were modeled as an incompressible, elastic membrane with a 5 × 5 stiffness matrix and wall thickness, *h*. The theory of small deformations superimposed on large was used to linearize the material stiffness around the mean pressure using *in vivo* axial and circumferential stretches (Baek et al., 2007; Cuomo et al., 2019). For both species, anisotropic stiffness parameters varied spatially for the aorta and main branches.

For the humans, thickness *h* and *in vivo* axial stretch were adopted from the literature: *h* was assumed to be 14% of the luminal radius for the young adult and 16% for the old adult (Roccabianca et al., 2014). Spatially varying parameters for the aorta were assigned for the ATA, DTA, and IAA, as described in section “Aortic Biomechanics.” Linearization of stiffness occurred at the mean diameter and mean pressure point of the pressure diameter curve. Branching vessels were assigned the stiffness of the closest aortic segment; for example, the upper branches were assigned the stiffness of the ATA.

For the mice, *h* and *in vivo* axial stretch were determined from *in vitro* testing (Ferruzzi et al., 2013). *In vivo* circumferential stretch was calculated from the unloaded diameter and pressurized diameter from μCT images. Linearization for the mice occurred at *in vivo* axial and circumferential stretches. For the ATA, *in vivo* axial stretch was determined from length in μCT images. Different values of stiffness and thickness were prescribed for six regions of the aorta, the ATA, proximal DTA (pDTA), distal DTA (dDTA), SAA, IAA, and one CCA based on *in vitro* testing. The same values for the four-fiber family parameters were used for both sections of the DTA, but because of differences in diameter, linearization was performed at different *in vivo* circumferential stretch for the proximal and distal portions. Upper branches (both CCAs, left subclavian, and right innominate artery) were assigned the same stiffness and thickness as the tested CCA. Middle branches (mesenteric, celiac, and left and right renal arteries) were assigned the same stiffness matrix as the SAA and the thickness of the CCA. Iliac and tail arteries were assigned the stiffness and thickness of the IAA.

Perivascular support given by stiffness (k_s_) and damping (c_s_) coefficients were applied to all vessels to represent the pressure (P_ext_) from tissues surrounding the vasculature (Moireau et al., 2012; Ferruzzi et al., 2018). In the human, different k_s_ and c_s_ were applied to the ATA, DTA, and IAA portions of the aorta, as described previously (Cuomo et al., 2017). Branches off the aorta were assigned the same values of perivascular support as the closest aortic segment. For the young adult human, k_s_ = 200 Pa/mm for the ATA, 100 Pa/mm for the DTA, and 10 Pa/mm for the IAA with c_s_ = 10 Pa^∗^s/mm for the entirety of the aorta. For the old adult human, k_s_ = 5,200 Pa/mm for the ATA, 5,050 Pa/mm for the DTA, and 10 Pa/mm for the IAA with c_s_ = 10 Pa^∗^s/mm for the entirety of the aorta. A similar approach was used for the mice, as described previously for the young adult (Cuomo et al., 2019). This resulted in applied k_s_ = 13,500 Pa/mm for the ATA, 9,000 Pa/mm for the pDTA, 16,500 Pa/mm for the dDTA, 20,000 Pa/mm for the SAA, 47,000 Pa/mm for the IAA, and 50,000 Pa/mm for the CCAs with c_s_ = 10 Pa^∗^s/mm for the young adult mouse; global values of k_s_ = 40 Pa/mm and c_s_ = 30 Pa^∗^s/mm were prescribed for the old adult mouse.

#### Pulse Wave Velocity Analysis

Pulse wave velocity was calculated from the ATA (near the aortic root) to the iliac artery (directly after the iliac bifurcation), which is different from the common carotid-femoral PWV (cfPWV) which fails to include the ATA, a region of particular change in aging (Redheuil et al., 2010). PWV was calculated as the ratio of the centerline distance between the ATA and iliac artery and the pulse transient time (PTT). To calculate the PTT, the foot of the pressure waves at each region of interest was calculated based on the “intersecting tangent algorithm” (Gaddum et al., 2013).

## Results

### Morphology

**FIGURE 3:**
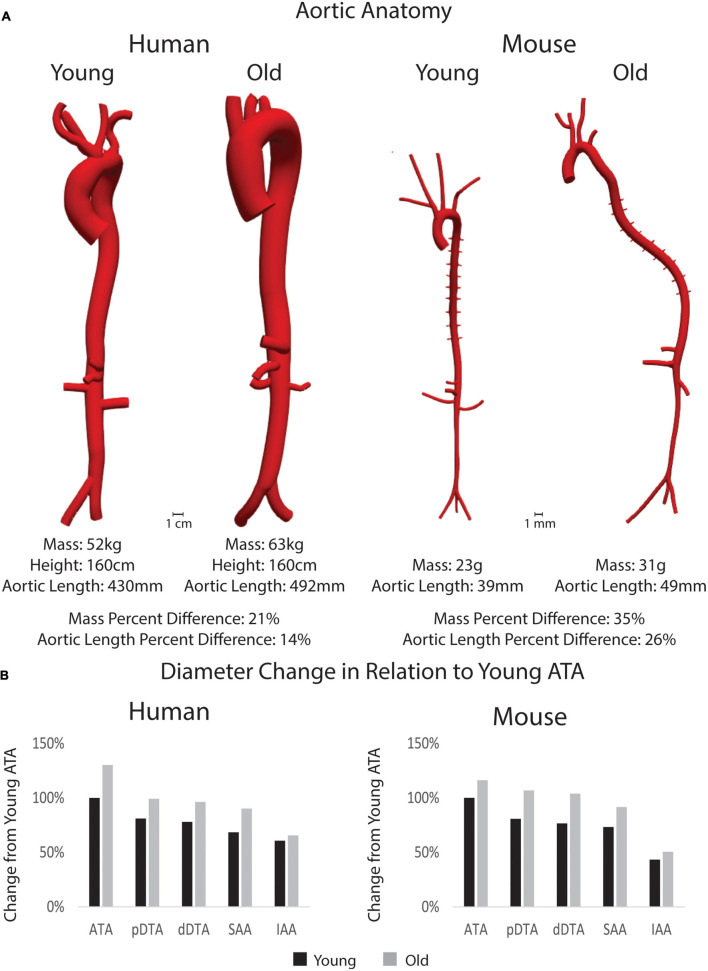
**(A)** 3D models of the aorta and main branches for each subject along with body mass, height (for humans), and aortic length. Percent differences between young and old adults for each species is shown for mass and aortic length. Anatomical models are shown in the same scale between age groups, but not between species. **(B)** Percent changes in inner diameter by region (pDTA, proximal descending thoracic aorta; dDTA, distal descending thoracic aorta; SAA, suprarenal abdominal aorta; and IAA, infrarenal abdominal aorta) of the aorta in comparison to the young ascending thoracic aorta (ATA_ diameter) for both species.

Anatomical models of the aorta and main branches of humans and mice can be seen in Figure 3A. Body mass, height, and aortic length for all subjects along with age-related percent differences are also presented. Quantitative differences between young adult and old adult aortic inner diameter are shown in the bar plots for Figure 3B, which show the percent diameter of each aortic region in comparison to the young ATA diameters. Humans experienced a more dramatic increase in aortic diameter than the mice, especially in the ATA though not in the IAA, noting that the aorta in the young human tapered gradually from the ATA to the IAA. This trend matches previously published work using human population data (Cuomo et al., 2017). Both young and adult mouse geometries revealed the drastic reductions in IAA diameter in relation to the other regions.

### Material Properties

**FIGURE 4:**
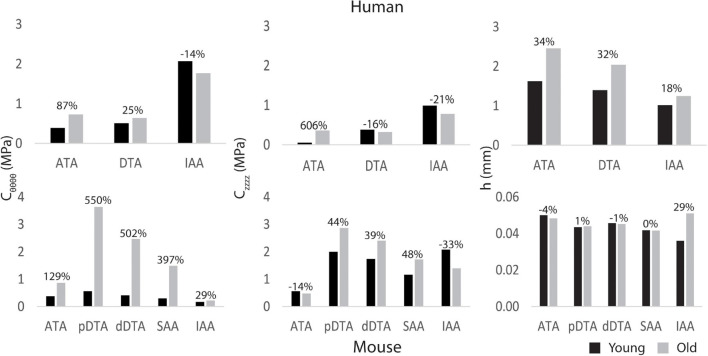
Regional circumferential (C_θ__θ__θ__θ_) and axial (C_zzzz_) material stiffness and wall thickness h for young adult and old adult humans (top) and mice (bottom) for five locations along the aorta (ATA, ascending thoracic aorta; pDTA, proximal descending thoracic aorta; dDTA, distal descending thoracic aorta; SAA, suprarenal abdominal aorta; and IAA, infrarenal abdominal aorta). The percentages indicate changes with aging relative to the young value. Note that the ATA stiffened the most with aging in humans (cf. Redheuil et al., 2010) whereas the DTA stiffened the most with aging in mice.

Figure 4 shows regional values of circumferential (C_θ__θ__θ__θ_) and axial (C_zzzz_) material stiffness and wall thickness (*h*) prescribed based on available data (ATA, DTA, and IAA for humans based on linearized values from literature; ATA, pDTA, dDTA, SAA, and IAA for mice based on biaxial tissue testing). Humans and mice demonstrated differing patterns of aortic stiffness. Human subjects exhibited increased material stiffness down the aorta with peak values occurring in the IAA. In contrast, mice exhibited peak circumferential stiffness in the pDTA that then decreased distally. Nevertheless, both species experienced an increase in circumferential stiffness with age in all aortic regions with the exception of the human IAA. This increase in stiffness was larger in the mouse than the human with the largest increase (550%) occurring in the mouse pDTA. Humans had the greatest increase in circumferential and axial stiffness (87 and 606%, respectively) from young to old in the ATA. For the mouse, axial stiffness increased with age in all aortic regions except for the IAA. Wall thickness increased in the human subjects with age, while in the mouse it remained relatively constant, except for in the IAA.

### Central and Peripheral Contributions of Resistance and Compliance

**FIGURE 5:**
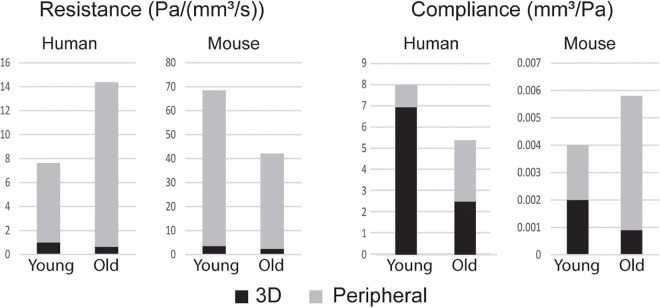
Total resistance R_TOT_ and compliance C_TOT_ broken into components of the central (3D) and peripheral vasculature.

Figure 5 shows the breakdown of R_TOT_ and C_TOT_ between the central (3D) and peripheral components. R_TOT_ was of similar magnitude for mice and humans, but C_TOT_ was three orders of magnitude smaller for the mice. The young adult human had smaller total resistance and larger total compliance than the older. Conversely, mice experienced a decrease in resistance and an increase in compliance with age. Humans had most of their total compliance in the central vasculature, while mice, particularly the older mice, had a majority in the periphery. For both species, there was a decrease in central and increase in peripheral compliance with age.

### Hemodynamics

**FIGURE 6:**
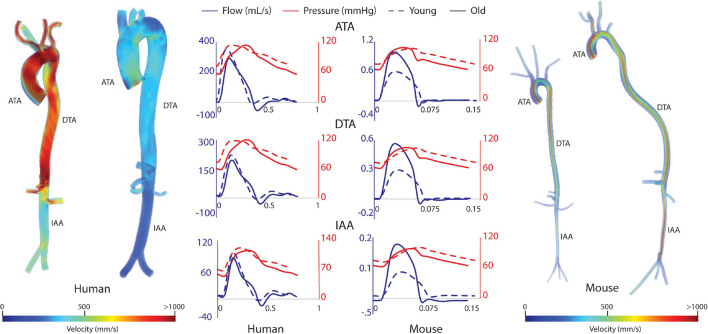
Volume rendering of blood velocity at peak systole for the humans (left) and mice (right), with pressure and flow waveforms at three sites along the aorta (ATA, ascending thoracic aorta; DTA, descending thoracic aorta; and IAA, infrarenal abdominal aorta) (center).

Computational results for peak systolic blood velocity as well as regional (ATA, DTA, and IAA) blood pressure and flow waveforms are shown in Figure 6 for all four basic models: young and old adult human, young and old adult mouse. The younger human had greater blood velocities than the older subject, consistent with the larger cardiac output (Figure 2) and smaller aortic dimensions (Figure 3). Conversely, the older mouse had greater blood velocities and higher aortic flows than the younger. Both older subjects show reverse flow in diastole, which is absent in the younger subjects. Human CO decreased 32% from the young to the old subjects, while mouse CO increased 52% with age.

**FIGURE 7:**
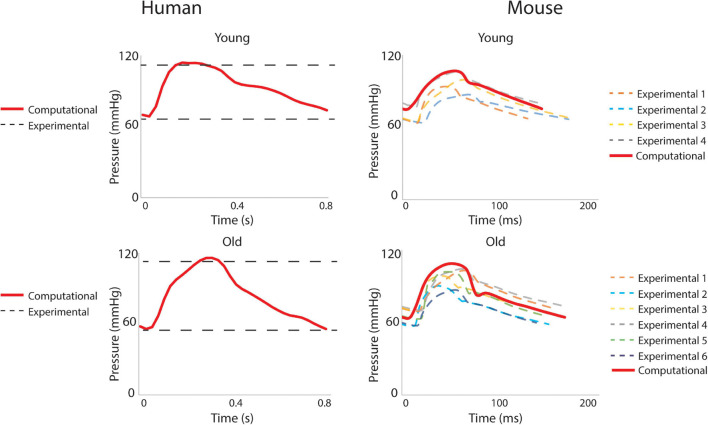
Validation of hemodynamic results using pressure data. For humans, the simulated left subclavian artery pressure (solid red line) compared well with systolic and diastolic pressure cuff values (dashed black lines). For the mice, simulated ascending thoracic aortic (ATA) flow waveforms (solid red lines) compared well with experimental data from multiple subjects (dashed lines).

Hemodynamic results were validated against experimental data. For the humans, averaged blood pressure cuff measurements were compared with the simulated (not imposed) left subclavian artery pressure (Figure 7), resulting in discrepancies less than 5% for diastolic and systolic pressures for both young and old adult humans. For the mice, simulated ATA pressure waveforms were compared with measured Millar ATA pressure waveforms in terms of pulse pressure, mean pressure, and slope of the diastolic decay (Figure 7). Further, simulated IAA and CCA mean flows showed good agreement with their experimental counterparts.

**FIGURE 8:**
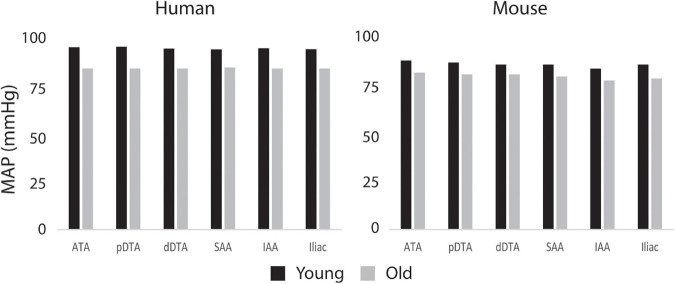
Computed values of mean arterial pressure (MAP) for five aortic locations (ATA, ascending thoracic aorta; pDTA, proximal descending thoracic aorta; dDTA, distal descending thoracic aorta; SAA, suprarenal abdominal aorta; and IAA, infrarenal abdominal aorta) and an iliac artery for all four primary study groups, all showing modest changes proximal to distal.

**FIGURE 9:**
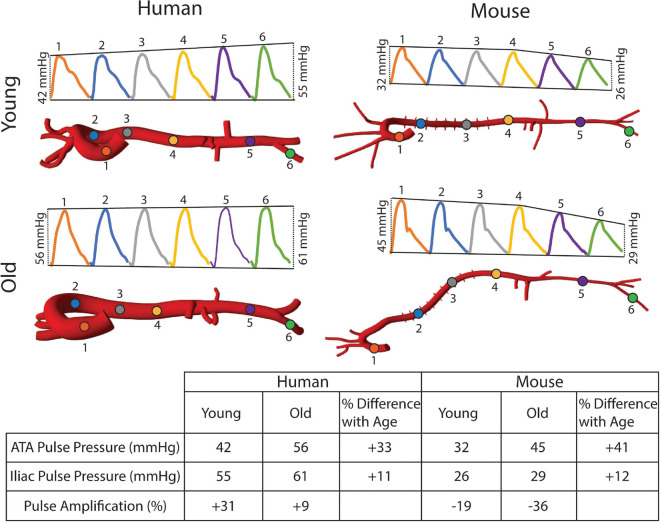
Pulse pressure amplification manifests in the human aorta, but not the mouse.

**FIGURE 10:**
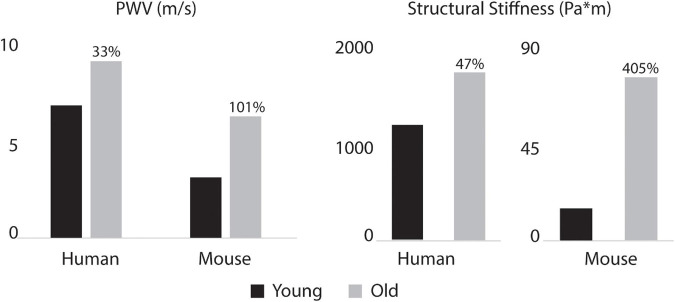
Aorto-iliac PWV **(left)** and spatially weighted averages of structural aortic stiffness **(right)** for the humans and mice. Percent difference from young to old are indicated for both species. Baseline values for the young subjects were 6.9 m/s for human and 3.2 m/s for mouse PWV and 1195.7 and 15.3 for human and mouse structural stiffness.

Figure 8 shows the calculated mean arterial pressure for five aortic locations (ATA, pDTA, dDTA, SAA, and IAA) and iliac artery. Mean pressure was similar between species and the pressure gradient down the aorta was small for all subjects. Figure 9 shows pressure waveforms at the same six locations. Numerical values for pulse pressure are indicated at the ATA and iliac artery. The overall spatial trends of the pressure waveforms differ significantly between species. Humans show an amplification of the pulse pressure down the aorta (31% increase for the young, 9% increase for the old, see table in Figure 9), consistent with the increase in material stiffness reported in Figure 4. On the other hand, mice show an attenuation of pulse pressure down the aorta (-19% for the young, -36% for the old), consistent with the reported decrease in material stiffness in the descending thoracic and infrarenal aorta (Figure 4). Conversely, both species show similar changes in ATA pulse pressure with aging: 33% increase for the old human and 41% increase for the old mouse. These increases in pulse pressure are driven by the similar stiffening of the ATA with age (87% increase in circumferential stiffness for the human, 129% increase for the mice, see Figure 4). Lastly, ATA-to-iliac PWV and spatially weighted averages of structural aortic stiffness can be seen in Figure 10. The humans had higher values of PWV than the mice. Yet, the mice showed a larger increase in PWV with age than did humans, consistent with the larger increase in structural stiffness.

## Discussion

Notwithstanding all that has been learned about vascular biology, mechanics, and physiology via the use of diverse animal models, mice have emerged as the primary model of choice in contemporary vascular research, including studies of the effects of hypertension and aging on the cardiovascular system (Paneni et al., 2017; Lerman et al., 2019). Nevertheless, similarities and differences in central arterial hemodynamics have not been studied in depth between humans and mice, especially in relation to aging. Here, we used consistent biomechanical assessments of central artery mechanical properties (species and age specific four-fiber family constitutive descriptions) and consistent FSI modeling to elucidate possible structurally induced differences in hemodynamics.

The representative healthy human subjects in this study showed a decrease in CO with aging, which is consistent with trends found in the literature. Katori (1979) measured resting cardiac output in a cohort of 105 healthy individuals and found a decrease in CO of 0.45 L/min per decade for individuals 20 and older. In contrast, our subjects showed an effective decrease in CO of 0.28 L/min per decade. Consistent reporting of changes in CO and other hemodynamic parameters with age in mice has been challenging due to variations in strains and experimental procedures (Doevendans et al., 1998). Here, mice showed an increase in CO with age which can be explained by the increase in body size (Ferruzzi et al., 2018). Furthermore, both older subjects had reverse flow throughout the aorta, which is absent in the younger subjects. This is of particular importance in the IAA, as reverse flow negatively impacts renal function (Hashimoto and Ito, 2015).

In this study, older subjects showed larger values of aortic length and diameter for both species. In the human, this is consistent with trends found in the literature (Craiem et al., 2012; Rylski et al., 2014). For the representative subjects used herein, aortic length increased more in the mice than in the humans because of the increased aortic tortuosity and body size in the older mouse. Aortic tortuosity often increases with age in humans as well (Cuomo et al., 2017; Ciuricã et al., 2019), although this was not seen in the old adult human subject in this study. Although not considered here, aortic tortuosity has also been reported to increase in mouse models of compromised elastic fiber integrity (e.g., *Fbln5*^–/–^), which capture some aspects of aortic aging in humans (Weiss et al., 2020). There was a trend toward a decreasing aortic inner diameter along the length of the aorta in both species, which is consistent with results reported in previous studies (Roccabianca et al., 2014; Cuomo et al., 2017).

Humans and mice showed marked differences in both spatial patterns of aortic stiffness and changes in stiffness with aging. Mainly, we see an increase in stiffness down the aorta in humans, which is consistent with trends found in literature (Mitchell, 2009; Roccabianca et al., 2014). In contrast, in mice the circumferential stiffness increases from the ATA to the pDTA, then decreases distally, which is consistent with previous murine studies (Bersi et al., 2017; Cuomo et al., 2019). With age, both species exhibit an increase in aortic stiffness. In the humans, the largest increase in stiffness with age occurs in the ATA. In the mouse, the largest stiffening happens in the DTA and SAA. These age-related changes in stiffness of the two species are most likely determined by different mechanisms: mechanical fatigue-induced loss of elastic fiber integrity over many decades in the human (Davis, 1993; Greenwald, 2007), and accumulation of proteoglycans and remodeled collagen in the mouse (Fleenor et al., 2010; Ferruzzi et al., 2018).

The aforementioned differences in aortic stiffness between species and age groups also resulted in marked hemodynamic differences. Consistent with previously reported findings (Safar and Laurent, 2003; O’Rourke and Hashimoto, 2007), humans present a pulse pressure amplification down the aorta while mice have pulse pressure attenuation down the aorta. However, both species showed an increase in ATA pulse pressure with age, which suggests similar hemodynamic responses to aging. In the humans, ATA and iliac pulse pressures became more similar with age, also consistent with prior findings (Safar and Laurent, 2003; O’Rourke and Hashimoto, 2007), whereas these metrics became more different in the mouse with age. Both species showed an increase in ATA to iliac PWV with age. Despite not commonly being used clinically due to difficulty in measurement, aortoiliac PWV has advantages over cfPWV, mainly that it includes the ATA, an area known for stiffening with age (Mitchell, 2009; Redheuil et al., 2010). For the humans, PWV values were consistent with mean healthy population data (Mattace-Raso et al., 2010) for both age groups: 6.2 ± 1.5 m/s (compared to our 6.9 m/s) for 30-year-old, and 10.9 ± 5.4 m/s (compared to our 9.23 m/s) for subjects >70-year-old subjects. While ATA to iliac PWV was directly calculated from our computational models, Mattace-Raso et al. (2010) measured cfPWV and scaled it by 0.8 to represent the ATA to iliac values.

Humans and mice also exhibited different trends in terms of RTOT and compliance. Figure 5 shows that most of the vascular compliance is in the periphery in mice, as opposed to in the central vasculature in humans (Saouti et al., 2010). The younger human has lower total resistance and larger total compliance than the older, which contradicts the trend seen in the mouse of a decrease in resistance and an increase in compliance with age. These findings in mice are consistent with previous computational FSI studies of hemodynamics in fibulin-5 deficient mice (Cuomo et al., 2019). Despite an overall decrease in vascular resistance and increase in vascular compliance, there is an increase in resistance and decrease in compliance in the central vasculature, which is consistent with our age-related changes in vascular diameter and stiffness.

Despite the importance and advantages of direct comparisons across species and age using consistent methods, limitations herein include the use of subject-specific morphological and hemodynamic data for the humans (noting expectation of considerable differences across subject cohorts) and population-based data for the mice. In this work, we do not present any population-based statistics. Statistical significance would be important to address for different patient cohorts or for different mouse groups, but with greater numbers of subjects. The key focus here is not on group-to-group differences, but rather on similarities and differences between representative subjects of the species, human and murine in particular. Furthermore, in both species, non-linear material properties were population based. All results were for females, and cardiovascular aging likely differs for males. Similar studies comparing young and old males and contrasting aged males and females would be interesting as well. Because we used population-based data for a particular mouse colony, our results hold for the C57BL/6 × 129/SvEv strain, with expected differences for pure C57BL/6 and 129/SvEv mice (cf. Spronck et al., 2021). Previous work has characterized regional aortic biomechanics in multiple mouse models, though without associated FSI analyses of the central hemodynamics (Ferruzzi et al., 2013; Bersi et al., 2017; Murtada et al., 2020; Spronck et al., 2020). It was beyond the present scope to consider effects of specific genetic mutations or modifiers in either the mice or the humans. Moreover, because we used subject-based data for the human, these results are not indicative of the entire population, although the subjects were selected such that changes in cardiac output, aortic dimensions, and aortic stiffness with age were consistent with reported population data. The only notable difference is the lack of increased tortuosity in the older human subject aorta. Furthermore, the *in vivo* data were collected under resting conditions in the human and under anesthesia in the mice. Finally, because of the ability to use invasive procedures in murine subjects, there are intrinsic differences in the measurement techniques between the species. To mitigate the effects of this, efforts were made to ensure the consistency of data. Particularly, despite not being able to perform biaxial tissue testing in humans, the four-fiber family model was used based on literature values and adapted to simulated biaxial test data (Roccabianca et al., 2014). Computational techniques were consistent between the species.

## Conclusion

To our knowledge, this study is the first comparison of the differences in hemodynamics, wall properties, and vascular anatomy as a function of age between mice and humans. We identified key differences between the human and murine central vasculature such as patterns in aortic stiffness, pulse amplification, amount and distribution of arterial resistance and compliance, and changes in CO, stiffening, and resistance and compliance with age, which question many uses of naturally aged mouse models for understanding certain aspects of cardiovascular aging in humans. Conversely, there are also key similarities between the species, as, for example, a global increase in aortic stiffness, increase in ATA pulse pressure, decrease in mean arterial pressure, reverse flow in older subjects, and increase in PWV. Furthermore, genetic manipulation of mouse models may lessen the differences by capturing broader population diversity, which is a critical step toward personalized medicine. For example, there are mouse models that effect elastin structure and content (Yanagisawa and Wagenseil, 2020), which may prove to be a more closely related analog for human aging.

## Data Availability Statement

The raw data supporting the conclusions of this article will be made available by the authors, without undue reservation.

## Ethics Statement

The studies involving human participants were reviewed and approved by the University of Michigan Board of Review. The patients/participants provided their written informed consent to participate in this study. The animal study was reviewed and approved by the Institutional Animal Care and Use Committee (IACUC) of Yale University, New Haven, CT, United States.

## Author Contributions

FC, NB, JH, and CF: conceptualization. FC, JF, and NB: data collection. SH, FC, JF, SR, JH, and CF: formal analysis. JH and CF: funding acquisition, supervision, and overall responsibility. SH and CF: writing of original draft. SH, FC, JF, NB, SR, JH, and CF: writing—review and editing and final approval of the manuscript. All authors contributed to the article and approved the submitted version.

## Conflict of Interest

The authors declare that the research was conducted in the absence of any commercial or financial relationships that could be construed as a potential conflict of interest.

## Publisher’s Note

All claims expressed in this article are solely those of the authors and do not necessarily represent those of their affiliated organizations, or those of the publisher, the editors and the reviewers. Any product that may be evaluated in this article, or claim that may be made by its manufacturer, is not guaranteed or endorsed by the publisher.

## Footnotes

1
jax.org
